# *CYP2C19* genotype and platelet aggregation test-guided dual antiplatelet therapy after off-pump coronary artery bypass grafting: A retrospective cohort study

**DOI:** 10.3389/fcvm.2022.1023004

**Published:** 2022-12-06

**Authors:** Haoyi Yao, Kaijie Qin, Yun Liu, Yi Yang, Jiaxi Zhu, Anqing Chen, Zhe Wang, Xiaofeng Ye, Mi Zhou, Haiqing Li, Jiapei Qiu, Qiang Zhao, Yunpeng Zhu

**Affiliations:** Department of Cardiovascular Surgery, Ruijin Hospital, Shanghai Jiao Tong University School of Medicine, Shanghai, China

**Keywords:** dual antiplatelet therapy (DAPT), *CYP2C19* genotype, platelet aggregation test (PAgT), off-pump coronary artery bypass grafting (OPCAB), major adverse cardiovascular events (MACE), major bleeding

## Abstract

**Background:**

Dual antiplatelet therapy (DAPT) is recommended in patients undergoing off-pump coronary artery bypass graft surgery (OPCAB). Clopidogrel is less effective among patients with loss-of-function (LoF) of *CYP2C19* alleles, while ticagrelor has direct effects on P2Y_12_ receptor. Whether a *CYP2C19* genotype plus platelet aggregation test (PAgT)-guided DAPT after CABG could improve clinical outcomes remain uncertain.

**Materials and methods:**

From August 2019 to December 2020, 1,134 consecutive patients who underwent OPCAB received DAPT for 1 year after surgery in Ruijin Hospital, Shanghai Jiao Tong University School of Medicine. According to the actual treatment they received in real-world, 382 (33.7%) of them received a traditional DAPT: aspirin 100 mg qd + clopidogrel 75 mg qd, no matter the *CYP2C19* genotype and response in platelet aggregation test (PAgT). The other 752 (66.3%) patients received an individual DAPT based on *CYP2C19* genotype and PAgT: aspirin 100 mg qd + clopidogrel 75 mg qd if *CYP2C19* was extensive metabolizer, or moderate metabolizer but normal response in PAgT; aspirin 100 mg qd + ticagrelor 90 mg bid if *CYP2C19* was poor metabolizer, or moderate metabolizer but no or low response in PAgT. One-year follow-up was achieved for all patients. The primary outcome was major adverse cardiovascular events (MACE), a composite of cardiovascular death, myocardial infarction, and stroke. The safety outcome was thrombolysis in myocardial infarction (TIMI) criteria major bleeding.

**Results:**

Compared with the traditional DAPT group, the risk of MACE in the individual DAPT group was significantly lower (5.5 vs. 9.2%, HR 0.583; 95% CI, 0.371–0.915; *P* = 0.019), mainly due to the decreased risk of MI (1.7 vs. 4.2%, HR 0.407; 95% CI, 0.196–0.846; *P* = 0.016). The risk of TIMI major bleeding events was similar between the two groups (5.3 vs. 6.0%, RR 0.883; 95% CI, 0.537–1.453; *P* = 0.626).

**Conclusion:**

For patients who underwent OPCAB, individual DAPT (*CYP2C19* genotype plus PAgT-guided strategy) was associated with a lower risk of MACE and a similar risk of major bleeding.

## Introduction

Dual antiplatelet therapy (DAPT) is defined as the combined application of aspirin and P2Y_12_ receptor inhibitor and is highly recommended for patients undergoing OPCAB (1). Currently, clopidogrel is the most widely used P2Y_12_ receptor inhibitor in patients undergoing PCI or OPCAB. As clopidogrel is a prodrug that requires inactivation by *CYP2C19*, its genetic variants could affect the conversion of clopidogrel, implying that the benefits of clopidogrel may be attenuated in patients with these genetic variants (2, 3). The metabolic activity of clopidogrel could be absent or decreased in carriers of *CYP2C19* loss-of-function (LOF) alleles (4–6). Conversely, ticagrelor is a direct-acting P2Y_12_ inhibitor that reversibly inhibits adenosine diphosphate (ADP)-mediated platelet aggregation (7). A comparison of the pharmacokinetics of ticagrelor and clopidogrel reveals that ticagrelor shows a stronger antiplatelet effect with less variation between patients and maintains better therapeutic uniformity (6–8).

Off-pump coronary artery bypass grafting (OPCAB) is the most effective and durable choice for ischemic heart disease. However, patients who have undergone OPCAB are still at risk of subsequent ischemic events and the development of graft dysfunction. Therefore, secondary prevention after OPCAB plays an important role in keeping graft patency and preventing major adverse cardiovascular events (MACE). Post-operation antiplatelet therapy is the most important among all the secondary preventions (1). It is unequivocally accepted that administration of aspirin after OPCAB is necessary; however, whether patients would benefit from clopidogrel remains controversial (9–18).

Currently, guidelines recommend ticagrelor prior to clopidogrel as first choice P2Y_12_ inhibitor in STEMI patients (19, 20). *CYP2C19* gene detection is an excellent tool for the selection of appropriate P2Y12 receptor inhibitors, which may improve the outcomes in patients with acute coronary syndrome (ACS) (21, 22). However, there have been few studies related to personalizing DAPT in patients undergoing CABG and recommended international guidelines are unavailable. On the other hand, epidemiological studies have confirmed that East Asians have a considerably higher frequency of *CYP2C19* LOF alleles than other races (23). However, clear evidence regarding the clinical benefit of individualized antiplatelet therapy based on *CYP2C19* genotype in Asians is lacking.

Currently, there is no exact conclusion on whether ticagrelor is safer and more effective than clopidogrel in patients undergoing OPCAB. Therefore, we conducted a retrospective cohort study to investigate whether an individual DAPT strategy based on *CYP2C19* genotype can obtain a better prognosis within 1 year in patients undergoing OPCAB in China.

## Materials and methods

### Patients

This single-center, non-randomized, retrospective cohort study was performed at the Ruijin Hospital, Shanghai Jiao Tong University School of Medicine. The study involved a total of 1,134 consecutive patients who underwent OPCAB between August 2019 to December 2020 and received DAPT for 1 year after surgery. This study was reviewed and approved by Ruijin Hospital Ethics Committee, Shanghai Jiao Tong University School of Medicine.

### *CYP2C19* genotyping

All patients underwent *CYP2C19* gene testing at the time of admission for the following variant alleles: CYP2C19*2 (rs4244285) and CYP2C19*3 (rs4986893).

According to the clinical pharmacogenetics implementation consortium (24), we classified the two-LoF-alleles-carriers as poor metabolizer (*2/*2, *2/*3, *3/*3), one-LoF-allele-carriers as moderate metabolizer (*1/*2, *1/*3), and non-LoF-allele-carriers as extensive metabolizer (*1/*1).

### Monitoring the platelet aggregation rate

We monitored the platelet aggregation rate using light transmission aggregation (LTA, 4 μmol/L ADP induced) at the time of admission, pre-operation and every alternate day from the first to seventh day after operation. We judged the therapeutic response to the medicine and formulated individual therapeutic schedule based on platelet aggregation test (PAgT). Platelet aggregation of < 30% was considered normal response, platelet aggregation > 60% was considered no response, while 30–60% were categorized as low response.

### P2Y_12_ inhibitor treatment

As a retrospective cohort study, actual antiplatelet treatment the patients received in real-world were depended on surgeons’ advice and patient’s compliance. At that period, *CYP2C19* gene testing and platelet function testing were already routine in our center, but surgeons were not required to formulate a unified antiplatelet therapy regimen based on the test results. Due to the difference in surgeons’ philosophy, we were surprised to find two completely different types of antiplatelet strategies and therefore this non-randomized retrospective study was conducted.

The patients who received a 75 mg dose of clopidogrel daily no matter the *CYP2C19* genotype and response in platelet aggregation test (PAgT), were allocated as the traditional DAPT group. The patients who received a 75 mg dose of clopidogrel daily only if *CYP2C19* was extensive metabolizer, or moderate metabolizer but normal response in PAgT; otherwise switched to a 90 mg dose of ticagrelor twice daily if *CYP2C19* was poor metabolizer, or moderate metabolizer but no or low response in PAgT, were allocated as the individual DAPT group.

Throughout the entire follow-up period, all the patients were administrated with a100 mg dose of aspirin daily. Pantoprazole or lansoprazole instead of omeprazole and esomeprazole were recommended to prevent gastrointestinal bleeding events.

### Study outcomes

The primary outcome was MACE, defined as a composite of cardiovascular death (CV death), myocardial infarction (MI) and stroke. Secondary outcomes included the individual components of MACE (CV death, MI, and stroke), all-cause death, non-CV death. Other outcomes included the variation in platelet aggregation rate in PAgT, and grafts outcome at 1-year post-CABG.

Grafts’ outcome was classified according to FitzGibbon grade criterion. Grade A was defined as excellent patency or stenosis < 50%, Grade B was stenosis ≥ 50% and Grade O was total occlusion.

Safety outcomes were major bleeding events using TIMI criteria, including CABG-related and non-CABG-related major bleeding events (25).

### Follow-up

According to local clinical practice protocol, all patients were encouraged to receive outpatient follow-up at 1 month, 3 months, 6 months, and 1-year post-CABG. If outpatient follow-up was not feasible, a telephone interview would be conducted. Graft outcome was assessed using multislice computed tomographic angiography at 1-year post-CABG. All these follow-up data were recorded in a local database.

### Statistical analysis

Statistical analysis of the baseline characteristics and outcomes were performed using IBM SPSS version 26.0 (IBM Corp., Armonk, NY, USA). Categorical variables were presented as numbers and percentages and compared using chi-square or Fisher’s exact tests. Continuous variables with normal distribution were expressed as mean ± standard deviation (± S.D), and differences between groups were analyzed using Student’s *t*-test.

Primary and secondary endpoints were compared using log-rank test. Kaplan-Meier curves and were performed using Prism version 8.0 (GraphPad Software, San Diego, CA, USA). Cox proportional hazard models were applied to calculate hazard ratios with 95% confidence intervals. A two-sided test was performed, and *P* value < 0.05 were considered statistically significant.

## Results

### Flow chart

Of the total 1,134 patients in this retrospective cohort study, 382 (33.7%) received a 75 mg dose of clopidogrel daily added to a 100 mg dose of aspirin, no matter the *CYP2C19* genotype and result of PAgT, were allocated as the traditional DAPT group.

**FIGURE 1:**
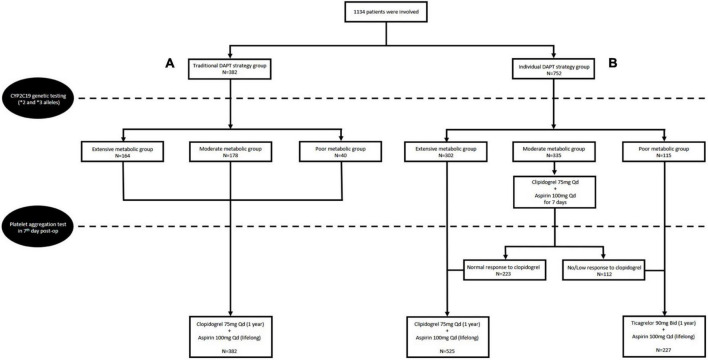
Flow chart of the study design and the traditional dual antiplatelet therapy (DAPT) group **(A)** and individual DAPT group **(B)**.

The other 752 (66.3%) patients received either a 75 mg dose of clopidogrel daily (525 patients) or a 90 mg dose of ticagrelor twice daily (227 patients) according to the guidance of *CYP2C19* genotype and PAgT, were allocated as the individual DAPT group. Of them, 302 patients with extensive metabolizer received a 75 mg dose of clopidogrel daily without a result of PAgT; 115 patients with poor metabolizer switched to a 90 mg dose of ticagrelor twice daily without a result of PAgT; 335 patients with moderate metabolizer received a 75 mg dose of clopidogrel daily for 1 week initially, and then underwent a PAgT; 233 patients with normal response continued clopidogrel treatment, while 112 patients with low response switched to ticagrelor treatment (Figure 1).

### Baseline characteristics

Among the 1,134 patients, 72.0% were men with a mean age of 62.7 ± 6.0 years. The overall prevalence of hypertension, diabetes mellitus, and hyperlipidemia was 67.0, 32.5, and 54.1%, respectively. Old myocardial infarction was present in 203 (17.9%) patients and 72 (6.3%) patients had previous PCI. The mean left ventricular ejection fraction was 60.8 ± 6.0%. 230 (20.3%) patients were with left main coronary disease. All patients underwent OPCAB and the mean number grafts was 3.2 ± 1.0. In addition to DAPT, the usage of other secondary prevention medications were all high, including 95.5% with beta-blockers, 88.6% with angiotensin-converting enzyme inhibitor or angiotensin receptor blockers, 99.3% with statin.

There was no significant difference in the baseline characteristics between the two groups (*P* > 0.05; Table 1).

**TABLE 1:** Demographics and characteristics of the study population.

Characteristics	Individual DAPT group (*N* = 382)	Traditional DAPT group (*N* = 382)	*P* value
Male, N (%)	545 (72.5%)	272 (71.2%)	0.65
Age, years, mean (SD)	62.5 ± 5.9	63.2 ± 6.2	0.10
Smoking, N (%)	348 (46.3%)	162 (42.4%)	0.22
**Medical history, N (%)**			
Hypertension	498 (66.2%)	261 (68.3%)	0.48
Diabetes mellitus	249 (33.1%)	119 (31.2%)	0.51
Hyperlipidemia	419 (55.7%)	195 (51.0%)	0.14
Myocardial infarction	142 (18.9%)	61 (16.0%)	0.23
Stroke	18 (2.4%)	10 (2.6%)	0.82
Renal insufficiency	59 (7.8%)	28 (7.3%)	0.76
Peripheral vascular disease	50 (6.6%)	18 (4.7%)	0.19
Preoperative LVEF, %, mean (SD)	61.0 ± 5.9	60.6 ± 6.2	0.45
Atrial fibrillation	63 (8.4%)	24 (6.3%)	0.21
PAgT, %, mean (SD)	42.2 ± 17.9	42.5 ± 19.0	0.81
**Previous revascularization, N (%)**			
PCI	46 (6.1%)	26 (6.8%)	0.65
**Coronary artery lesions, N (%)**			
Left main coronary disease	156 (20.7%)	74 (19.4%)	0.59
Diffuse vascular disease	79 (10.5%)	32 (8.4%)	0.25
Average number of graft, mean (SD)	3.20 ± 0.96	3.23 ± 0.95	0.62
**Medical treatment, N (%)**			
Beta-blockers	716 (95.2%)	367 (96.1%)	0.51
ACEI and ARB	674 (89.6%)	331 (86.6%)	0.14
Statin	746 (99.2%)	380 (99.5%)	0.60
Calcium channel inhibitor	215 (28.6%)	125 (32.7%)	0.15
Proton pump inhibitor	752 (100.0%)	382 (100.0%)	1.00

### Distribution of *CYP2C19* genotypes and platelet aggregation rate variation

All 382 patients in the traditional DAPT group underwent *CYP2C19* genotyping. A total of 164 (42.9%) patients were non-carriers of LOF alleles while the remaining 218 (57.1%) patients carried LOF alleles, including 178 (46.6%) patients with one LOF allele and 40 (10.5%) patients with two LOF alleles. The proportion of non-carriers, one-LOF-allele-carriers, and two-LOF-alleles-carriers in the individual DAPT group were 302 (40.2%), 335 (44.5%), and 115 (15.3%), respectively. The data of PAgT showed that out of all the patients in the traditional DAPT group, only 61.0% had normal response to clopidogrel and only 5.0% of patients who carried two LOF alleles had normal response. In the traditional DAPT group the response to clopidogrel significantly varied between the patients with different *CYP2C19* genotypes (*P* = 0.000; Table 2A); however, the differences were partly reversed in individual DAPT group after using ticagrelor in the poor metabolizer patients (*P* = 0.000; Table 2B).

**TABLE 2:** Analysis of metabolic type of *CYP2C19* in traditional dual antiplatelet therapy (DAPT) group (A) and individual DAPT group (B).

A	Extensive metabolizer (*N* = 164)	Moderate metabolizer (*N* = 178)	Poor metabolizer (*N* = 40)	Total (*N* = 382)
Normal response (%)	120 (73.2)	111 (62.3)	2 (5.0)	233 (61.0)
Low response (%)	40 (24.4)	32 (18.0)	6 (15.0)	78 (20.4)
No response (%)	4 (2.4)	35 (19.7)	32 (80.0)	71 (18.6)

**B**	**Extensive metabolizer (*N* = 302)**	**Moderate metabolizer (*N* = 335)**	**Poor metabolizer (*N* = 115)**	**Total (*N* = 752)**

Normal response (%)	216 (71.5)	223 (66.6)	66 (57.4)	505 (67.2)
Low response (%)	77 (25.5)	51 (15.2)	43 (37.4)	171 (22.7)
No response (%)	9 (3.0)	61 (18.2)	6 (5.2)	76 (10.1)

**FIGURE 2:**
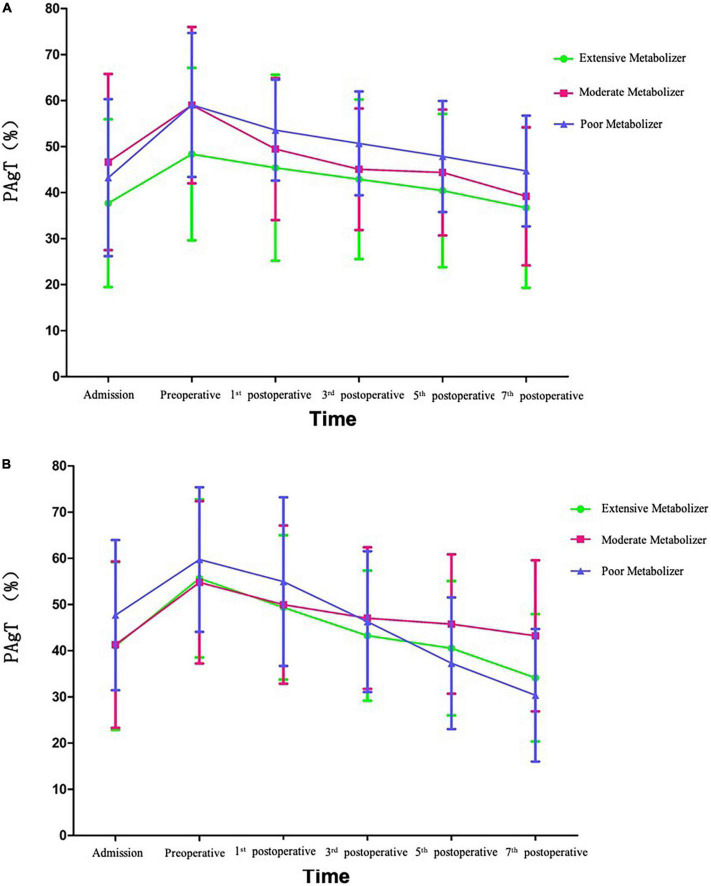
Tendency of platelet aggregation rate in traditional dual antiplatelet therapy (DAPT) group **(A)** and individual DAPT group **(B)**.

Platelet aggregation test (PAgT) was monitored at admission, pre-operation, and once every other day from the first to seventh day after operation. The poor metabolizer patients in individual DAPT group had a crossover with the extensive and moderate metabolizer groups and had promising effect on restraining platelet aggregation (Figure 2).

### Clinical outcomes

**FIGURE 3:**
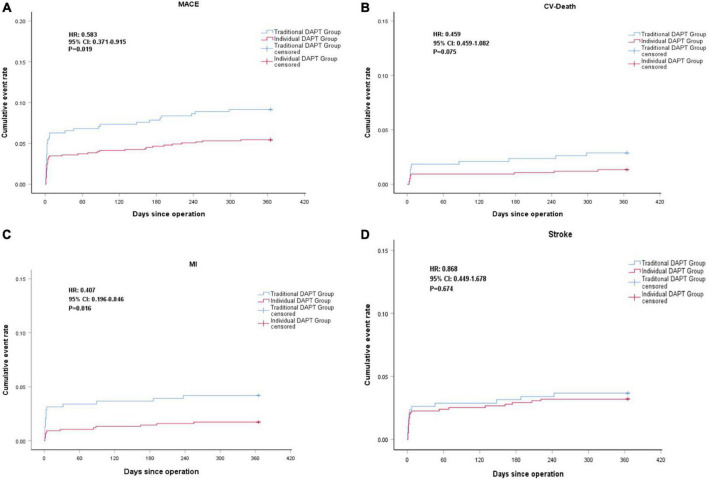
Kaplan-Meier curve of major adverse cardiovascular events (MACE) **(A)**, Cardiovascular (CV)-Death **(B)**, MI **(C)**, Stroke **(D)** during the 1 year follow-up.

There was no loss to follow-up in either group. During the follow-up period, MACE was observed in 76 (6.7%) patients, including 41 cases in the individual DAPT group and 35 cases in the traditional DAPT group. Compared to the traditional DAPT group, the risk of MACE in the individual DAPT group was significantly lower (5.5 vs. 9.2%, HR 0.583; 95% CI, 0.371–0.915; *P* = 0.019), mainly due to the decreased risk of MI (1.7 vs. 4.2%, HR 0.407; 95% CI, 0.196–0.846; *P* = 0.016). The risk of all-cause death was numerically lower in the individual DAPT group when compared with the traditional DAPT group (2.3 vs. 4.2%, HR 0.534; 95% CI, 0.270– 1.058; *P* = 0.072), which might mainly due to the decreased risk of CV death (1.3 vs. 2.9%, HR 0.459; 95% CI, 0.195–1.082; *P* = 0.075). On the other hand, no significant differences were found in the risk of non-CV death (0.9 vs. 1.3%, HR 0.709; 95% CI, 0.225–2.234; *P* = 0.557), ischemic stroke (3.1 vs. 3.4%, HR 0.896; 95% CI, 0.454–1.770; *P* = 0.753), and hemorrhagic stroke (0.1 vs. 0.3%, HR 0.507; 95% CI, 0.032–8.111; *P* = 0.631) between groups (Figure 3; Table 3).

**TABLE 3:** Endpoints of individual dual antiplatelet therapy (DAPT) group vs. traditional DAPT group.

End point	Individual DAPT group (*N* = 752)	Traditional DAPT group (*N* = 382)	Hazard ratio (95% CI)	*P* value
			
	No. of patients (%)			
MACE	41 (5.5)	35 (9.2)	0.583 (0.371–0.915)	0.019
All-cause death	17 (2.3)	16 (4.2)	0.534 (0.270–1.058)	0.072
CV death	10 (1.3)	11 (2.9)	0.459 (0.195–1.082)	0.075
Non-CV death	7 (0.9)	5 (1.3)	0.709 (0.225–2.234)	0.557
MI	13 (1.7)	16 (4.2)	0.407 (0.196–0.846)	0.016
Stroke	24 (3.2)	14 (3.7)	0.868 (0.449–1.678)	0.674
Ischemic stroke	23 (3.1)	13 (3.4)	0.896 (0.454–1.770)	0.753
Hemorrhagic stroke	1 (0.1)	1 (0.3)	0.507 (0.032–8.111)	0.631

Major bleeding events were observed in a total of 63 patients (5.6%), including 40 cases in the individual DAPT group and 23 cases in the traditional DAPT group. The risk of overall major bleeding events was similar between the groups (5.3 vs. 6.0%, RR 0.883; 95% CI, 0.537–1.453; *P* = 0.626) (Table 4).

**TABLE 4:** Safety endpoints of individual dual antiplatelet therapy (DAPT) group vs. traditional DAPT group.

End point	Individual DAPT group (*N* = 752)	Traditional DAPT group (*N* = 382)	Risk ratio (95% CI)	*P* value
			
	No. of patients (%)			
CABG-relate major bleeding	25 (3.3)	16 (4.2)	0.794 (0.429–1.469)	0.461
Re-operative stanch	11 (1.5)	8 (2.1)	0.698 (0.283–1.722)	0.434
Fatal bleeding	2 (0.3)	1 (0.3)	1.016 (0.092–11.169)	1.000
Blood transfusion>5U in 48h or drainage volume>2L in 24h	12 (1.6)	7 (1.8)	0.871 (0.346–2.194)	0.769
Non-CABG-related major bleeding	15 (2.0)	7 (1.8)	1.089 (0.448–2.647)	0.852
Overall major bleeding	40 (5.3)	23 (6.0)	0.883 (0.537–1.453)	0.626

The risk of MACE between the genotypes was not significantly different in the traditional DAPT group (extensive metabolism vs. non-extensive metabolism, 5.5 vs. 11.9%, HR 0.511; 95% CI, 0.246–1.065; *P* = 0.073) or in the individual DAPT group (extensive metabolism vs. non-extensive metabolism, 5.6 vs. 5.3%, HR 1.050; 95% CI, 0.564–1.954; *P* = 0.878). We observed a decreased risk of MACE in patients with non-extensive metabolism, which might be amplified when sample size is increased (Tables 5, 6). Safety analyses showed that the incidence of major bleeding events in traditional DAPT group and in individual DAPT group between genotypes was not significantly different (*P* values > 0.05) (Supplementary Tables 1, 2).

**TABLE 5:** Endpoints of individual dual antiplatelet therapy (DAPT) group (genotype-guided).

End point	Extensive metabolism group (*N* = 302)	Non-extensive metabolism group (*N* = 450)	Hazard ratio (95% CI)	*P* value
			
	No. of patients (%)			
MACE	17 (5.6)	24 (5.3)	1.050 (0.564–1.954)	0.878
All-cause death	9 (3.0)	8 (1.8)	1.686 (0.650–4.369)	0.283
CV death	5 (1.7)	5 (1.1)	1.494 (0.433–5.162)	0.525
Non-CV death	4 (1.3)	3 (0.7)	1.996 (0.447–8.916)	0.366
MI	5 (1.7)	8 (1.8)	0.932 (0.305–2.848)	0.901
Stroke	10 (3.3)	14 (3.1)	1.057 (0.469–2.379)	0.894
Ischemic stroke	9 (3.0)	14 (3.1)	0.950 (0.411–2.195)	0.904
Hemorrhagic stroke	1 (0.3)	0 (0.0)	/	0.585

**TABLE 6:** Endpoints of traditional dual antiplatelet therapy (DAPT) group (genotype-guided).

End point	Extensive metabolism group (*N* = 164)	Non-extensive metabolism group (*N* = 218)	Hazard ratio (95% CI)	*P* value
			
	No. of patients (%)			
MACE	10 (5.5)	25 (11.9)	0.511 (0.246–1.065)	0.073
All-cause death	5 (3.0)	11 (5.0)	0.595 (0.207–1.714)	0.336
CV death	3 (1.8)	8 (3.7)	0.494 (0.131–1.862)	0.297
Non-CV death	2 (1.2)	3 (1.4)	0.884 (0.148–5.289)	0.892
MI	4 (2.4)	12 (5.5)	0.435 (0.140–1.349)	0.149
Stroke	5 (3.0)	9 (4.1)	0.730 (0.244–2.177)	0.572
Ischemic stroke	4 (2.4)	9 (4.1)	0.582 (0.179–1.890)	0.368
Hemorrhagic stroke	1 (0.6)	0 (0.0)	/	0.590

### Grafts outcomes

A total of 336 of 382 patients in the traditional DAPT group and 679 of 752 patients in the individual DAPT group underwent multislice computed tomographic angiography 1 year after surgery. There was no significant difference in patency of ITA and RA between the two groups and the patency of SVG in traditional DAPT group was significantly lower than that in the individual DAPT group (84.4 vs. 91.4%, OR 0.506; 95% CI, 0.387–0.661; *P* = 0.000) (Table 7).

**TABLE 7:** Grafts patency between traditional dual antiplatelet therapy (DAPT) group and individual DAPT group.

	Traditional DAPT group (*N* = 336)			Traditional DAPT group (*N* = 679)			Odds ratio (95% CI)	*P* value
	FitzGibbon A (%)	FitzGibbon B + O (%)	Total	FitzGibbon A (%)	FitzGibbon B + O (%)	Total		
Internal thoracic artery (ITA)	314 (97.8)	7 (2.2)	321	640 (98.2)	12 (1.8)	652	0.841 (0.328–2.157)	0.718
Radial artery (RA)	17 (94.4)	1 (5.6)	18	40 (95.2)	2 (4.8)	42	0.850 (0.072–10.015)	1.000
Saphenous vein (SVG)	636 (84.4)	118 (15.6)	754	1354 (91.4)	127 (8.6)	1481	0.506 (0.387–0.661)	< 0.001

## Discussion

To the best of our knowledge, there are few studies on individualized pharmacogenomic antiplatelet therapy in patients after CABG, particularly in the Chinese population. Our study demonstrated that individualized antiplatelet therapy strategy based on *CYP2C19* genotypes and PAgT monitoring can significantly reduce the risk of MACE and MI in patients within 12 months after OPCAB. Although only 1015 (89.5%) patients underwent multislice computed tomographic angiography 1 year after surgery, we observed a significantly increased patency rate of vein grafts in the individual DAPT group. Safety analysis revealed that the individual DAPT group had the similar risk of major bleeding as the traditional DAPT group.

Clopidogrel, a prodrug metabolized by *CYP2C19*, inhibits diphosphate-induced platelet aggregation, platelet cycloxygenase-1, and interrupts thromboxane A2 formation. Compared with aspirin alone, clopidogrel combined with aspirin has a stronger synergistic antithrombotic effect. A combination of aspirin and clopidogrel can effectively reduce the risk of graft failure and MACE among patients undergoing CABG, which suggests that this population may benefit from intensive secondary prevention (12, 15). According to the recent guidelines from ACC/AHA 2015, aspirin plus clopidogrel is the recommended standard medical therapy after OPCAB (I/A) (1). Despite the overall benefit of clopidogrel, some individuals may be less responsive to it (26).

Ticagrelor has shown a higher efficacy than clopidogrel in patients with acute coronary syndrome (ACS) and is a promising new antiplatelet agent (7). Several studies have confirmed that ticagrelor is superior to aspirin in maintaining vein grafts patency and preventing MACE within 1 year after CABG (27, 28). But there was also study found that both aspirin plus ticagrelor, and aspirin plus clopidogrel can maintain a fairly high graft patency rate and *CYP2C19* genotypes may have no obvious effect on graft patency during the 1 year after CABG (29). In the platelet inhibition and patient outcomes (Plato) study and its further hoc subgroup study of CABG, the addition of ticagrelor with low-dose aspirin in patients significantly reduced overall mortality and cardiovascular mortality (4.7 vs. 9.7%, ticagrelor versus clopidogrel, *P* < 0.01; 4.1 vs. 7.9%, ticagrelor versus clopidogrel, *P* < 0.01) without an increase in CABG-related major bleeding (hazard ratio for ticagrelor group vs. clopidogrel group, 1.01; 95% CI, 0.90–1.15; *P* = 0.84) (30). According to the recent guidelines of ACC/AHA 2015, aspirin plus ticagrelor (preferred over clopidogrel) is the recommended standard medical therapy after CABG in ACS populations (IIa/B) (1).

The proportion of *CYP2C19* LOF allele carriers in our study (57.1% in the traditional DAPT group and 59.8% in the individual DAPT group) was similar to that previously reported in Asians and significantly higher than in western populations (21, 31). A higher risk of MACE among LOF allele carriers than non-carriers in the PCI population treated with clopidogrel was reported previously (32, 33). Based on available evidence, it is reasonable that DAPT strategies in Asians needs to be considered prudently because of ethnic differences in *CYP2C19* allele frequencies. A large meta-analysis showed that *CYP2C19* genotype was significantly associated with clopidogrel response but not with the risk of MACE (34). Additionally, another study reported that the level of platelet reactivity is not always associated with clinical outcomes in patients with ACS after PCI (35). Most studies are focused on patients undergoing PCI and to the best of our knowledge, there are no existing studies on patients undergoing CABG, especially in Asians. We found significant advantages of individualized therapy in individual DAPT group in terms of MACE and vein graft patency. This suggests that the use of ticagrelor, particularly in Chinese patients undergoing OPCAB, is meaningful.

In our study, we considered the *CYP2C19* genotype and the reaction of platelet aggregation to clopidogrel for selecting P2Y_12_ receptor inhibitors. In our study, 33.4% (112/335) moderate metabolizer patients (one-LOF-allele carriers) in individual DAPT group were switched from clopidogrel to ticagrelor because they had low or no response to clopidogrel treatment for 7 days after surgery. The safety study showed that there was no significant difference in the risk of bleeding events between the two groups. Based on the results of this study, we demonstrated that our individual DAPT strategy considering both *CYP2C19* genotype and platelet aggregation monitoring might help in achieving better outcomes without an increased risk of bleeding.

In view of the widespread use of clopidogrel worldwide, the high frequency of *CYP2C19* gene mutations in Asians may lead to futility of antiplatelet therapy in patients after CABG. Unfortunately, a well-designed DAPT strategy based on *CYP2C19* genotyping is not widely adopted. The FDA black-box warning recommends avoiding *CYP2C19* poor metabolizers with clopidogrel, but mandates *CYP2C19* genetic testing (36). The 2011 ACCF/AHA/SCAI PCI and 2014 ACC/AHA NSTE-ACS guidelines address the role of platelet function testing and genetic testing in patients receiving DAPT (20, 37). Unfortunately, platelet function and *CYP2C19* genetic testing are not recommended for routine use because no RCT was conducted to explore whether they could improve the outcomes (38). Although this was a retrospective cohort study with weak evidence-based medicine, to some extent, our study findings may provide evidence for *CYP2C19* genetic testing and platelet function testing among Asian patients undergoing OPCAB.

Our study had several limitations. First, as a retrospective cohort study, the antiplatelet treatment the patients received were depended on physician’s advice and patient’s compliance rather than randomized allocation, which would bring selection bias. Secondly, we did not detect frequency of *CYP2C19* allele *17 variants as the mutation takes place in the intron and we did not have appropriate detection means. Third, this study was a single-center study and the sample size was still not large enough. Fifth, nearly all the patients were of Han ethnicity, which might not be representative of the characteristics of the Asian population. Considering the positive results of our study, it is meaningful to carry out a multicenter, randomized, prospective, and blinded trial to verify the benefits of the individual DAPT strategy.

## Conclusion

Compared with a traditional DAPT strategy (aspirin plus clopidogrel), an individual DAPT strategy with *CYP2C19* genotype plus PAgT-guided (switched to aspirin plus ticagrelor if necessary) was associated with a lower risk of MACE and a similar risk of major bleeding in Chinese patients within 1 year after OPCAB.

## Data availability statement

The original contributions presented in the study are included in the article/Supplementary material, further inquiries can be directed to the corresponding authors.

## Ethics statement

The studies involving human participants were reviewed and approved by the Ruijin Hospital Ethics Committee Shanghai Jiao Tong University School of Medicine. Written informed consent for participation was not required for this study in accordance with the national legislation and the institutional requirements.

## Author contributions

HY and YZ: conception and design and administrative support. YL: determination of clinical events. HY, YY, and JZ: collection and upload of data. HY and KQ: data analysis and interpretation. HY, KQ, and YZ: manuscript writing. AC, ZW, XY, MZ, HL, JQ, QZ, and YZ: critical reading and revision. All authors contributed to the article and approved the submitted version.
