# Supplementary material for: CYP2C19 genotype and platelet aggregation test-guided dual antiplatelet therapy after off-pump coronary artery bypass grafting: A retrospective cohort study
Source: Front Cardiovasc Med. 2022 Dec 6;9:1023004. doi: 10.3389/fcvm.2022.1023004 (PMC9766355; doi:10.3389/fcvm.2022.1023004)
Supplement: Supplementary file 1 [file Data_Sheet_1.docx]

Supplementary Material

# Supplementary Tables

**Supplementary Table 1.** Safety Endpoints of Individual DAPT Group (genotype-guided).

| **End Point** | **Extensive Metabolism Group (N=302)** | | **non-Extensive Metabolism Group (N=450)** | **Risk Ratio (95% CI)** | **P Value** |
| --- | --- | --- | --- | --- | --- |
|  |  |  |  |  |  |
|  |  |  |  |  |  |
|  | no. of patients (%) | | |  |  |
|  |  | | |  |  |
| CABG-relate major bleeding | | 13(4.3) | 12(2.7) | 1.614(0.747-3.490) | 0.219 |
| Re-operative stanch | | 5(1.7) | 6(1.3) | 1.242(0.382-4.032) | 0.763 |
| Fatal bleeding | | 1(0.3) | 1(0.2) | 1.490(0.094-23.731) | 1.000 |
| Blood transfusion>5U in 48h or drainage volume>2L in 24h | | 7(2.3) | 5(1.1) | 2.086(0.668-6.512) | 0.239 |
|  |  |  |  |  |  |
| Non-CABG-related major bleeding | | 24(7.9) | 27(6.0) | 1.325(0.779-2.251) | 0.298 |
| Overall major bleeding | | 37(12.2) | 39(8.7) | 1.414 (0.924–2.163) | 0.110 |

**Supplementary Table 2.** Safety Endpoints of Traditional DAPT Group (genotype-guided).

| **End Point** | **Extensive Metabolism Group (N=164)** | | **non-Extensive Metabolism Group (N=218)** | **Risk Ratio (95% CI)** | **P Value** |
| --- | --- | --- | --- | --- | --- |
|  |  |  |  |  |  |
|  |  |  |  |  |  |
|  | no. of patients (%) | | |  |  |
|  |  | | |  |  |
| CABG-relate major bleeding | | 8(4.9) | 8(3.7) | 1.329(0.510-3.468) | 0.560 |
| Re-operative stanch | | 4(2.4) | 4(1.8) | 1.329(0.337-5.236) | 0.729 |
| Fatal bleeding | | 1(0.6) | 0(0.0) | / | 0.429 |
| Blood transfusion>5U in 48h or drainage volume>2L in 24h | | 3(1.8) | 4(1.8) | 0.997(0.226-4.393) | 1.000 |
|  |  |  |  |  |  |
| Non-CABG-related major bleeding | | 11(6.7) | 10(4.6) | 1.462(0.636-3.360) | 0.368 |
| Overall major bleeding | | 19(11.6) | 18(8.3) | 1.403 (0.761–2.588) | 0.276 |
